# Spatio-temporal occurrence, burden, risk factors and modelling methods for estimating scrub typhus burden from global to subnational resolutions: a systematic review protocol

**DOI:** 10.12688/wellcomeopenres.18533.1

**Published:** 2022-11-24

**Authors:** Qian Wang, Benn Sartorius, Nicholas Philip John Day, Richard James Maude

**Affiliations:** 1 Mahidol Oxford Tropical Medicine Research Unit, Faculty of Tropical Medicine, Mahidol University, Bangkok, Thailand; 2 Centre for Tropical Medicine and Global Health, Nuffield Department of Medicine, University of Oxford, Oxford, UK; 3 Department of Health Metrics Sciences, School of Medicine, University of Washington, Seattle, Washington, USA; 4 Harvard TH Chan School of Public Health, Harvard University, Boston, USA

**Keywords:** scrub typhus, systematic review, burden, incidence, prevalence, risk factor, model

## Abstract

**Background:** Scrub typhus is a neglected life-threatening vector-borne disease mainly caused by the bacterium *Orientia tsutsugamushi*, which is occasionally transmitted to humans during feeding of larval mites. It has been estimated that more than 1 billion persons potentially threatened and 1 million clinical cases occur annually across the world; however, it is unclear how this estimate was computed (and what the original source was) and much remains unknown regarding its global burden and risk factors. This systematic review aims to provide a comprehensive overview of the spatial-temporal distribution of scrub typhus, associated burden and risk factors at global, national and subnational resolutions, and to review the burden estimation models used at those different scales.

**Methods:** A systematic search for literature on scrub typhus occurrence, risk factors and modelling methods will be conducted. PubMed and five other databases will be searched for published literature, and Google Scholar and nine other databases will be used to search for grey literatures. All titles/abstracts of the searched records will be separately assessed by two reviewers, who will then screen the full-text of potential records to decide eligibility. A pre-formatted spreadsheet will be used by one reviewer to extract data from qualifying research, with a second reviewer checking the results. Data will be tabulated, synthesized descriptively, and summarized narratively for each review question. Where appropriate, meta-analyses will be conducted. The risk of bias will be assessed, and potential publication bias will be detected.

**Discussion:** This review will provide a comprehensive understanding of the current occurrence, spatial-temporal distribution, and burden of scrub typhus, identify associated risk factors from global to subnational resolutions, consolidate the best practice modeling framework(s) to estimate the burden of scrub typhus at various geographic/temporal resolutions, and decompose the relative contributions of various risk factors at scale.

**PROSPERO Registration:** CRD42022315209

## Abbreviations

CNKI: China National Knowledge Infrastructure; WHO: World Health Organization; ARDS: acute respiratory distress syndrome; MODS: multi-organ dysfunction syndrome; PRISMA: Preferred Reporting Items for Systematic Reviews and Meta-Analyses; DALY: Disability-adjusted life year; YLD: years lost due to disability; OR: odds ratios

## Introduction

Scrub typhus, also known as tsutsugamushi disease, is a serious febrile illness caused by the bacterium *Orientia tsutsugamushi* along with the closely related and recently described *Orientia chuto* and *chiloensis*. It is mostly spread by the larval stage of *Leptotrombidium* mites, commonly referred to as chiggers, to mammalian hosts, including humans ^1^.

The most common features in patients with scrub typhus are fever and headache ^2^, along with other manifestations of the infection such as eschars, regional lymphadenopathy, myalgia, tinnitus, rash, cough, and conjunctival suffusion ^3, 4^. Complications may include acute respiratory distress syndrome (ARDS), acute renal failure, septic shock, subacute thyroiditis, and multi-organ dysfunction syndrome (MODS) ^2, 3, 5, 6^. Mortality in severe cases can reach 30–70% ^7^, with approximately 6% untreated scrub typhus patients and 1.4% in infected individuals dying despite treatment ^8^. The lack of further distinct symptoms, coupled with a general reliance on serological tests, makes it difficult to distinguish scrub typhus from other common febrile illnesses, which makes scrub typhus ‘probably one of the most underdiagnosed and underreported febrile illnesses requiring hospitalization in the region’ ^9^.

The endemic area of scrub typhus was traditionally believed to be restricted to a well-defined geographic region called the “tsutsugamushi triangle”, extending from Russia far east in the north, northern Australia in the south, the southwestern Pacific islands in the east, and Afghanistan and Pakistan in the west ^10^. Many countries in the region have long been reported as suffering from scrub typhus, such as China, Japan, Thailand, and India ^11– 14^. Recently, however, scrub typhus has continued to spread in endemic areas and has also expanded geographically ^15^. With the increase in human activities, human cases and/or animal infections are starting to appear in countries previously considered non-endemic, in the Middle East, Africa, South America and Europe, where it may represent an emerging infectious disease ^16– 20^. The increase in incidence and the geographic expansion of the range of cases underscores the likely underestimation of this disease.

It has been estimated that more than 1 billion persons are under potential threat of disease and 1 million clinical cases occur annually across the world ^21^; however, it is unclear how this estimate was computed (and what the original source was) and much remains unknown regarding the disease’s global burden and risk factors. Scrub typhus has been a neglected disease and its current global distribution is highly uncertain. Therefore, this review aims to provide a comprehensive view of the global, national and sub-national scrub typhus burden over time, the role of different factors in the transmission and promotion of scrub typhus at various geographical scales, as well as the best practice modelling or estimation frameworks to quantify these metrics and the contribution of contextually relevant risk factors. We hope this review will result in more attention to this neglected disease and to establish the research agenda for producing new generalizable information to bridge gaps in comprehension of the burden of scrub typhus.

## Protocol

This systematic review adheres to PRISMA (Preferred Reporting Items for Systematic Reviews and Meta-Analyses) guidelines, and this protocol is being reported in accordance with PRISMA-P guidelines.

### Objectives

A systematic review will be conducted with the following objectives for scrub typhus globally: 1. determine the incidence and/or prevalence at different geographic scales and for different time periods; 2. identify the associated burden *e.g.* mortality, disability-adjusted life years (DALYs) or years lost due to disability (YLDs); 3. identify risk factors for occurrence; 4. identify mathematical or statistical methods or models which have been applied (and performed best) to predict occurrence or estimate burden; and 5. decompose the relative contributions of various risk factors at scale to identify which provide the most predictive power within these methodological frameworks to predict occurrence and estimate burden at global, national and subnational resolutions.

### Definitions

Outcome measures of interest for scrub typhus include: 1. prevalence of test positivity, reported numbers of cases or incidence rate; 2. reported fatalities and attributable mortality rates, DALYs, YLDs; 3. risk factors for occurrence, including biological ( *e.g.* vector and host density), meteorological ( *e.g.* temperature), geographical ( *e.g.* land use), socio-economic ( *e.g.* income, gross domestic product), behavioral ( *e.g.* outdoor activity and protective clothing use), and comorbidities such as autoimmune diseases; 4. modelling methods including the type of model developed and used to estimate the relationship between the risk factors and scrub typhus, and to predict associated occurrence and estimate burden such as DALYs; and 5. subset of risk factors with greatest predictive power.

### Inclusion criteria

1. Time-period: no time restriction

2. Language: no language restriction

3. Study type: observational studies, case reports and series, surveillance studies/reports, clinical trials

4. Population: people at risk of scrub typhus; people with scrub typhus test positivity in prevalence surveys; and scrub typhus patients meeting case definition (with a febrile illness confirmed by a molecular/serological diagnostic test)

5. Outcome: occurrence (incidence, prevalence); burden (mortality, DALY and/or YLD due to scrub typhus)

6. Studies that give risk factors for scrub typhus occurrence or burden estimation

7. Studies that use mathematical or statistical methods or models to predict occurrence and/or estimate the burden or relationship between risk factors and scrub typhus.

### Exclusion criteria

1. Studies whose complete texts are unavailable online or from the respective author.

2. Studies with smaller samples when duplicates or studies published in more than one report are found.

3. Case definition not clearly defined or not applied consistently.

4. Correspondence, animal studies, experimental studies, and test evaluation/validation studies.

### Search strategy

A systematic search strategy will be applied to search for potentially eligible studies in appropriate databases, including PubMed, Scopus, Ovid Medline, Ovid Embase, Web of Science, and CNKI. The potential grey literatures will be identified using the appropriate search strategy in the following data sources: Google Scholar, NY Academy of Medicine Grey Literature Report, MedNar, WHO Global Index Medicus, ProQuest Dissertations, Theses Global database, Preprints in Europe PMC, WHO International Clinical Registry Platform, ClinicalTrials.gov, and the ProMED website.

The search terms will be the combination of the name of scrub typhus and the outcome words. For English databases, the proposed search term will be used: ("scrub typhus" OR tsutsugamushi OR "mite typhus" OR "japanese river fever" OR "Orientia tsu" OR “Rickettsia tsu” OR "Akamushi disease" OR mijtekoorts OR "tropical typhus" OR "mite borne rickettsiosis" OR "mite borne typhus") AND (epidemiolog* OR frequency OR incidence* OR morbidit* OR occurrence OR prevalence OR probability OR rate OR statistic* OR burden OR distribution OR risk OR case*). For the Chinese database, CNKI, the search terms will be: (“恙虫病” OR “丛林斑疹伤寒”) AND (“分布” OR “时空” OR “流行” OR “发生” OR “负担” OR “风险” OR “病例”).

If any of the searches return any additional pertinent keywords, we will update the electronic search strategy to include them and document the modifications. Review studies will have their reference lists scanned for relevant articles.

### Study records

The initial search results will be pooled, and duplicates removed using EndNote 20, with the source, search strategies, date of search and received records number tracked and recorded.

Two reviewers will independently screen the titles/abstracts of identified records, and a third reviewer will resolve any disagreements in decisions. Rayyan software will be used to streamline this process. For articles written in other non-English languages, we will first use Google Translate to determine their likelihood of being eligible, and then contact potential native-speaking researcher collaborators to help screen them.

Two authors will use the results of title/abstract screening, obtain full text of publications potentially matching the eligibility criteria to do the second round of screening to decide their final eligibility.

All articles that finally meet the criteria will be appropriately and randomly divided into two groups. Two reviewers will independently perform corresponding data extraction and finally cross-check to avoid manual errors using designed standardized forms. Any disagreements will be resolved by a third reviewer. Authors will be contacted for missing data, and if no response is received within three weeks this will be repeated again. If no response is received, the data will be recorded as missing.

The standardized forms will be designed into two sheets to capture the primary results, one for occurrence and burden, and one for risk factors and models, depending on the focus of the review questions. The various parameters will be extracted by the authors utilizing the standard data extraction forms; the key data items included in these data extraction forms are:

•   Characteristics of the studies (including list of authors, year of publication, language of publication, country of first institution, geographical region of the study, year when the study started and ended and type of study design, name of website or resource used).

•   Characteristics of the study participants: age, gender, ethnicity and employment.

•   Diagnostic methods used.

•   Types of outcome measure: prevalence/incidence/deaths/number of cases, DALYs, YLDs, mortality.

•   Epidemiological outcomes: prevalence/incidence/number of cases/deaths due to scrub typhus, predicted/potential burden caused, geographic location (finest scale that can be found, such as latitude and longitude, village name, county name, district name), month and year of outcome measure.

•   Risk factors outcome: risk factor’s name, source, resolution and its associations/correlations with scrub typhus.

•   Method/model outcome: Volume and time-space scale of data used for modelling, Mathematical or statistical methods or models developed and used, method/model performance (accuracy, discrimination) and validation method.

### Data synthesis/charting

Data will be tabulated, synthesized descriptively, and summarized narratively in the submitted report for each review question. The means and standard deviations of the quantitative variables or the median and interquartile range will be presented and when applicable, qualitative variables in absolute and relative frequencies will also be demonstrated. Where appropriate, meta-analyses will be performed using the proper software, heterogeneity will be investigated using meta-regression and subgroup analyses will be considered ( *e.g.*, by type of diagnostic methods used). The goal of the meta-analysis is to calculate the incidence and prevalence of scrub typhus in regional and national scale in different years by calculating the pooled data. Based on the quality of evidence, types of study, diagnostic method used, and the sensitivity analysis will be done by omitting one study or one type of study at a certain time and recalculating the pooled prevalence and other results for the remaining studies in the systematic review.

For the occurrence of scrub typhus, incidence and prevalence will be quantified separately. Weighing the reported rates with the research sample sizes will be used to make a modification to the sample size. Descriptive spatiotemporal analyses will be carried out and thematic maps of the distribution of incidence and prevalence, and their trends by year, will be produced. When the number of studies allows for the estimation of between-study variation, a random effects model will be employed for the analysis. We will also conduct impact and outlier analysis to see how certain research affect our combined estimates of the prevalence of scrub typhus ^22^.

For the risk factors, descriptive summaries will be provided, and further statistical analyses will be performed for risk factors that have appeared in more than one article. Odds ratios (OR) values and 95% confidence intervals were calculated based on the number of participants and the corresponding number of cases in the included articles. For the heterogeneity analysis, we will examine risk factors that are included in more than five studies, and then look at the heterogeneity between the studies reporting the risk factor using Cochran’s Q-test. Furthermore, we will carry out a meta-regression for those risk factors that show considerable heterogeneity (≥75%). We will consider meta-regression and subgroup analyses to investigate sources of heterogeneity. The statistical software R extended by package “metafor” for meta-analysis will be used to perform the meta-analysis.

For the models, the data collected will be synthetized in descriptive summaries. Given that the studies included in this review will differ with population samples and geographical level, quantitative statistics will not be performed for the result of models. The better-performing methods and models will be selected in terms of whether and how sufficient validation (in-sample validation and out-of-sample validation) was done, how the best model was selected, and what metric was used.

Subgroup analysis will be carried out to investigate whether there are any differences in outcomes by study characteristics such as type of publication (grey literature versus peer-reviewed publication), language used (English versus others) and type of diagnostic methods used. We also plan to test differences according to sex, race/ethnicity, and age if suitable data are available. Risk factors and models where the number of primary studies is greater than ten will be explored to ensure statistical power.

### Critical appraisal

Based on the diversity of research types included in the articles, we will use an adjusted risk of bias assessment method to assess the methodological quality of the included studies and the Newcastle-Ottawa Quality Assessment Scale (NOS) ^23^ will be a reference. We will use this instrument to score each included study and then stratify into low, medium, and high quality. Evaluation of each article will be conducted independently by two reviewers and cross-checked, and any inconsistent results will be discussed with a third review for final decision.

The funnel plots will be applied to detect potential publication bias and small study effects. Egger’s method will be used to assess asymmetry and we will use a significance cut-off of p<0.05 as evidence of statistically significant publication bias ^24^.

### Ethics policies

This review is secondary research and does not require any ethics approval.

## Discussion

Through this systematic review, we aim to provide a more comprehensive assessment of scrub typhus occurrence and associated burden at global, national and subnational scales and to further draw attention to this neglected disease. The review will also attempt to identify potentially important risk factors associated with scrub typhus occurrence and burden, which would be useful for occurrence and burden prediction and how this varies across geography and scale. We think the results of this review will benefit a variety of stakeholders, including patients, public health officials, policymakers, and relevant researchers. In order to provide a clear, transparent and robust review, this protocol was developed so that further issues throughout the review process can be minimized, and if any crucial adjustments are determined to be necessary, they will be disclosed in the published review.

## Data Availability

No data are associated with this article. Zenodo: Spatio-temporal occurrence, burden, risk factors and modelling methods for estimating scrub typhus burden from global to subnational resolutions: a systematic review protocol- PRISMA-P checklist. https://doi.org/10.5281/zenodo.7299952 ^25^ Data are available under the terms of the Creative Commons Attribution 4.0 International license (CC-BY 4.0).
