# Supplementary material for: Spatio-temporal occurrence, burden, risk factors and modelling methods for estimating scrub typhus burden from global to subnational resolutions: a systematic review protocol
Source: Wellcome Open Res. 2024 Sep 12;7:285. Originally published 2022 Nov 24. [Version 2] doi: 10.12688/wellcomeopenres.18533.2 (PMC11443196; doi:10.12688/wellcomeopenres.18533.2)
Supplement: Peer review history

# Reviewer report: Reviewer response for version 2

**DOI:** 10.21956/wellcomeopenres.25381.r99213

**Published:** 2024-09-30

**Authors:** Benjamin L Makepeace

**Affiliations:** 1 University of Liverpool, Liverpool, England, UK

The authors have made some important changes to the inclusion and exclusion criteria and the search strategy that have greatly improved the review protocol. I consider all of my original concerns to have been addressed effectively.

Is the study design appropriate for the research question?

Yes

Is the rationale for, and objectives of, the study clearly described?

Yes

Are sufficient details of the methods provided to allow replication by others?

Partly

Are the datasets clearly presented in a useable and accessible format?

Not applicable

Reviewer Expertise:

NA

I confirm that I have read this submission and believe that I have an appropriate level of expertise to confirm that it is of an acceptable scientific standard.

# Reviewer report: Reviewer response for version 2

**DOI:** 10.21956/wellcomeopenres.25381.r99212

**Published:** 2024-09-14

**Authors:** Yuejin Liang

**Affiliations:** 1 The University of Texas Medical Branch Health, Galveston, Texas, USA

All issues have been addressed.

Is the study design appropriate for the research question?

Yes

Is the rationale for, and objectives of, the study clearly described?

Yes

Are sufficient details of the methods provided to allow replication by others?

Yes

Are the datasets clearly presented in a useable and accessible format?

Not applicable

Reviewer Expertise:

My research is focused on the host immune responses against Orientia infection. By using animal models, we aim to reveal the mechanisms of bacterial dissemination in early infection and how host innate immunity combatting with this process.

I confirm that I have read this submission and believe that I have an appropriate level of expertise to confirm that it is of an acceptable scientific standard.

# Reviewer report: Reviewer response for version 1

**DOI:** 10.21956/wellcomeopenres.20551.r91429

**Published:** 2024-08-26

**Authors:** Benjamin L Makepeace

**Affiliations:** 1 University of Liverpool, Liverpool, England, UK

This systematic review protocol describes a planned study to determine risk factors for scrub typhus acquisition and estimate its distribution and global burden. This is a critically important and overdue task, and an ambitious meta-analysis on a global scale is sorely needed by the scrub typhus research community in order to improve advocacy for research on this highly neglected condition. For the most part, the protocol is well constructed and fairly comprehensive, but I have some queries about the inclusion/exclusion criteria and the scope of the objectives.

**Introduction**

 I have one relatively minor comment on this section. The sentence “With the increase in human activities, human cases and/or animal infections are starting to appear in countries previously considered non-endemic, in the Middle East, Africa, South America and Europe, where it may represent an emerging infectious disease.” The ecoepidemiology of ST outside the tsutsugamushi triangle has not been studied yet in great depth, but this sentence implies it is a recent phenomenon linked to human expansion in the Anthropocene. While endemic ST outside the Asia-Pacific has only been confirmed molecularly this century, the divergence between the three *Orientia* species and the use of different genera of chigger vectors suggests these pathogens diverged from *O. tsutsugamushi* hundreds of thousands, if not millions, of years ago. Please rephrase this sentence. 

**Protocol**

**Inclusion criteria:**

 “1. Time-period: no time restriction.”

 This needs some justification for a study that presumably is aiming to estimate current ST burden, distribution and risk factors. It is debatable for instance if studies from the WWII period would be pertinent to any of these goals (arguably yes for risk factors, but as those data will be largely restricted to non-indigenous populations, it could be misleading in today’s context).

 “3. risk factors for occurrence, including biological (e.g. vector and host density).” 

 I note that “animal studies” are listed under exclusion criteria below. Perhaps laboratory animal studies are intended for exclusion here, not field studies? Non-human animals and vectors are the only hosts relevant to the Orientia lifecycle, and some studies combine human and non-human fieldwork elements, or incorporate historic or newly collected host/vector data to examine correlations with previously recorded human ST surveillance data. Clarification is required here. 

 “7. Studies that use mathematical or statistical methods or models to predict occurrence and/or estimate the burden or relationship between risk factors and scrub typhus.”

 There are many such studies that attempt this, and their reliability is dependent on the quality of the underlying ST case data, which for some endemic countries, has been recorded for decades in national surveillance systems. However, the underlying data may not be publicly available in all cases. Will the planned meta-analyses use national surveillance data where available, including from more recent years than existing publications may have covered?

Is the study design appropriate for the research question?

Yes

Is the rationale for, and objectives of, the study clearly described?

Yes

Are sufficient details of the methods provided to allow replication by others?

Partly

Are the datasets clearly presented in a useable and accessible format?

Not applicable

Reviewer Expertise:

Vector-borne diseases/zoonoses: genomic aspects, molecular ecoepidemiology, cell culture, vaccine and drug development.

I confirm that I have read this submission and believe that I have an appropriate level of expertise to confirm that it is of an acceptable scientific standard, however I have significant reservations, as outlined above.

# Response

**Published:** 2024-08-29

**Authors:** Qian Wang

**Affiliations:** Mahidol Oxford Tropical Medicine Research Unit, Thailand

**Reviewer comment: **I have one relatively minor comment on this section. The sentence “With the increase in human activities, human cases and/or animal infections are starting to appear in countries previously considered non-endemic, in the Middle East, Africa, South America and Europe, where it may represent an emerging infectious disease.” The ecoepidemiology of ST outside the tsutsugamushi triangle has not been studied yet in great depth, but this sentence implies it is a recent phenomenon linked to human expansion in the Anthropocene. While endemic ST outside the Asia-Pacific has only been confirmed molecularly this century, the divergence between the three Orientia species and the use of different genera of chigger vectors suggests these pathogens diverged from O. tsutsugamushi hundreds of thousands, if not millions, of years ago. Please rephrase this sentence.

**Response:** Thank you for your thoughtful comment. We agree that the original sentence could lead to misunderstandings about the ecoepidemiology of scrub typhus. To avoid this, we have removed the phrase "with the increase in human activities" from the sentence to better reflect the long-standing presence of *Orientia* species outside the tsutsugamushi triangle.

**Reviewer comment: **Protocol-Inclusion criteria:

 “1. Time-period: no time restriction.” This needs some justification for a study that presumably is aiming to estimate current ST burden, distribution and risk factors. It is debatable for instance if studies from the WWII period would be pertinent to any of these goals (arguably yes for risk factors, but as those data will be largely restricted to non-indigenous populations, it could be misleading in today’s context).

**Response:** Thank you for your insightful comment. We have chosen not to impose any language or time restrictions in our search strategy because we do not want to miss any potentially valuable information for this highly neglected disease. However, we recognize that older studies may not always be relevant. During the screening process, many older publications will likely be excluded if they lack a clear disease definition or do not use appropriate statistical models to assess risk factors. Additionally, for many countries, modern evidence is scarce or non-existent. In such cases, older studies may provide the only available evidence to support our understanding of scrub typhus in these regions, and we do not want to overlook these sources.

**Reviewer comment: **“3. risk factors for occurrence, including biological (e.g. vector and host density).” I note that “animal studies” are listed under exclusion criteria below. Perhaps laboratory animal studies are intended for exclusion here, not field studies? Non-human animals and vectors are the only hosts relevant to the Orientia lifecycle, and some studies combine human and non-human fieldwork elements, or incorporate historic or newly collected host/vector data to examine correlations with previously recorded human ST surveillance data. Clarification is required here.

**Response: **Thank you for your observation. We have clarified the exclusion criteria by specifying that only "animal studies without data related to human cases" will be excluded. This change ensures that relevant field studies involving non-human animals and vectors, which are crucial to the Orientia lifecycle, are included. We recognize that some studies combine human and non-human fieldwork or use host/vector data to examine correlations with human scrub typhus surveillance data, and these will be considered in our analysis.

**Reviewer comment: **“7. Studies that use mathematical or statistical methods or models to predict occurrence and/or estimate the burden or relationship between risk factors and scrub typhus.” There are many such studies that attempt this, and their reliability is dependent on the quality of the underlying ST case data, which for some endemic countries, has been recorded for decades in national surveillance systems. However, the underlying data may not be publicly available in all cases. Will the planned meta-analyses use national surveillance data where available, including from more recent years than existing publications may have covered?

**Response: **Thank you for your valuable feedback. We have addressed your concern by revising our search strategy to include the following: "Further efforts will be made to obtain and incorporate data from national surveillance systems where available." Additionally, in the data synthesis and charting section, we have added: "For incidence, we will integrate data from national disease surveillance systems and publications to understand regional and temporal patterns." Hope this will help ensure that our meta-analysis incorporates the most reliable and current data, allowing us to accurately assess the burden, distribution, and risk factors associated with scrub typhus.

# Reviewer report: Reviewer response for version 1

**DOI:** 10.21956/wellcomeopenres.20551.r61982

**Published:** 2023-07-20

**Authors:** Yuejin Liang

**Affiliations:** 1 The University of Texas Medical Branch Health, Galveston, Texas, USA

In this study, Wang *et al.*, generate a protocol for systemic review to investigate the current state of literatures on the spatial-temporal distribution of scrub typhus, associated burden and risk factors at global, national and subnational resolutions, and the burden estimation models used at those different scales. Scrub typhus is a life-threatening, but severely neglected vector-borne disease; however, its current global distribution is highly uncertain. This protocol is rigorous, methodic and outcome-oriented with high significance. My suggestions are below.

1. A recent study detected Orientia in field-collected free-living Eutrombicula chigger in US. This is the first time to find Orientia in North America. Please update the background in the manuscript and cite this paper: Chen *et al. *(2023 ^1^).
2. It is better to generate tables to summarize the criteria for study and the search strategy.

Is the study design appropriate for the research question?

Yes

Is the rationale for, and objectives of, the study clearly described?

Yes

Are sufficient details of the methods provided to allow replication by others?

Yes

Are the datasets clearly presented in a useable and accessible format?

Not applicable

Reviewer Expertise:

My research is focused on the host immune responses against Orientia infection. By using animal models, we aim to reveal the mechanisms of bacterial dissemination in early infection and how host innate immunity combatting with this process.

I confirm that I have read this submission and believe that I have an appropriate level of expertise to confirm that it is of an acceptable scientific standard.

## References

[1] : Detection of Orientia spp. Bacteria in Field-Collected Free-Living Eutrombicula Chigger Mites, United States. *Emerg Infect Dis.* .2023;29(8) : Reference source 10.3201/eid2908.230528PMC1037086637486323

# Response

**Published:** 2024-08-29

**Authors:** Qian Wang

**Affiliations:** Mahidol Oxford Tropical Medicine Research Unit, Thailand

**Reviewer comment:** A recent study detected *Orientia* in field collected free-living *Eutrombicula* chigger in US. This is the first time to find *Orientia* in North America. Please update the background in the manuscript and cite this paper: Chen et al. (20231).

**Response: **Thank you for providing this important reference. We have updated the introduction to include the following sentence: "Notably, a recent study detected *Orientia* in field-collected free-living chiggers in the United States, marking the first time *Orientia* has been found in North America." This addition helps to contextualize the significance of the study in the broader discussion of scrub typhus' geographic expansion.

**Reviewer comment: **It is better to generate tables to summarize the criteria for study and the search strategy.

**Response:** Thank you for your suggestion. We have generated tables summarizing the inclusion and exclusion criteria, as well as the search strategy, and have incorporated them into the revised manuscript (Table 1,2 and 3).
